# Magnetic NH_2_-MIL-101(Al)/Chitosan nanocomposite as a novel adsorbent for the removal of azithromycin: modeling and process optimization

**DOI:** 10.1038/s41598-022-21551-3

**Published:** 2022-11-08

**Authors:** Ali Azari, Mohammad Malakoutian, Kamyar Yaghmaeain, Neemat Jaafarzadeh, Nabi Shariatifar, Gholamabbas Mohammadi, Mahmood Reza Masoudi, Reza Sadeghi, Sanaz Hamzeh, Hossein Kamani

**Affiliations:** 1 Sirjan School of Medical Sciences, Sirjan, Iran; 2 Student Research Committee, Sirjan School of Medical Sciences, Sirjan, Iran; 3 Department of Environmental Health Engineering, School of Public Health, Kerman University of Medical Sciences, Kerman, Iran; 4 Department of Environmental Health Engineering, School of Public Health, Tehran University of Medical Sciences, Tehran, Iran; 5 Department of Environmental Health Engineering, School of Public Health, Jondishapour University of Medical Sciences, Ahvaz, Iran; 6 Health Promotion Research Center, Zahedan University of Medical Sciences, Zahedan, Iran

**Keywords:** Environmental sciences, Chemistry, Materials science, Mathematics and computing, Nanoscience and technology

## Abstract

In the present study, the magnetic NH_2_-MIL-101(Al)/chitosan nanocomposite (MIL/Cs@Fe_3_O_4_ NCs) was synthesized and used in the removal of azithromycin (AZT) from an aqueous solution for the first time. The as-synthesized MIL/Cs@Fe_3_O_4_ NCs was characterized by SEM, TEM, XRD, FTIR, BET, and VSM techniques. The effect of various key factors in the AZT adsorption process was modeled and optimized using response surface methodology based on central composite design (RSM-CCD). The low value of p-value (1.3101e−06) and RSD (1.873) parameters, along with the coefficient of determination > 0.997 implied that the developed model was well fitted with experimental data. Under the optimized conditions, including pH: 7.992, adsorbent dose: 0.279 g/L, time: 64.256 min and AZT concentration: 10.107 mg/L, removal efficiency and AZT adsorption capacity were obtained as 98.362 ± 3.24% and 238.553 mg/g, respectively. The fitting of data with the Langmuir isotherm (R^2^: 0.998, X^2^: 0.011) and Pseudo-second-order kinetics (R^2^: 0.999, X^2^: 0.013) showed that the adsorption process is monolayer and chemical in nature. ΔH° > 0, ΔS° > 0, and ∆G° < 0 indicated that AZT removal was spontaneous and endothermic in nature. The effect of Magnesium on AZT adsorption was more complicated than other background ions. Reuse of the adsorbent in 10 consecutive experiments showed that removal efficiency was reduced by about 30.24%. The performance of MIL/Cs@Fe_3_O_4_ NCs under real conditions was also tested and promising results were achieved, except in the treatment of AZT from raw wastewater.

## Introduction

In the last decades, the removal of antibiotics has received more attention from researchers due to their cumulative properties, high toxicity, chemical stability, biological activity, and the development of bacterial resistance even in small amounts^1^. So far, a variety of antibiotics including macrolides, tetracyclines, and chloramphenicols are found in environmental media^2^. Azithromycin (C_38_H_72_N_2_O_12_, AZT) is a broad-spectrum macrolide antibiotic with a long half-life that is primarily used to treat respiratory, enteric, and genitourinary infections^3,4^. The presence of azithromycin in a water body is usually related to personal hygiene products, pharmaceutical industry waste, hospital waste, therapeutic drugs, animal husbandry, agriculture, and aquaculture. The presence of trace amounts of AZT in drinking water is a public health concern because little is known about the potential chronic health effects associated with the long-term ingestion of mixtures of these compounds through drinking water^5^. Moreover, AZT antibiotic residues can alter the human microbiome and cause health disturbances, such as allergic reactions, chronic toxic effects after prolonged exposure, and disruption of digestive system functions^6^. On the other side, due to their extensive usage and negligible human metabolism, about 30–90% of AZT antibiotics are excreted unchanged in the body of humans and animals as active compounds in urine and feces^7,8^. Environmental exposure to active pharmaceutical ingredients (APIs) can have negative effects on the health of ecosystems and humans^9^. Therefore, effective removal of AZT antibiotics from wastewater before their discharge is inevitable and necessary. Conventional wastewater treatment processes are not usually able to remove pharmaceutical compounds^10^.

Among various wastewater treatment processes, adsorption is considered as a promising and widely-used technique for removing pharmaceutical substances from wastewater due to its flexible design, ease of operation, and cost-effectiveness^11,12^. Nevertheless, the development of affordable, high-performance and reusable adsorbents for the pharmaceutical wastewater treatment remains a challenge among researchers and requires more comprehensive studies. MOFs are organic–inorganic hybrid crystalline porous structures consist of metal complex or cluster joined by organic ligands. MOFs have unique properties such as large pore volumes, adjustable pore size, fine tunable pore surface property, low density, flexible framework and alterable pore functionalities, flexibility in chemistry design, pore size, shape, and structure and high affinity to polymers and environmental friendly^13,14^. As reported in the literature, MOFs are widely used in many fields such as electrocatalysis, electrochemical devices (fuel cells, electrolysis cells, batteries, supercapacitors, sensors, etc.), gas storage and separation, liquid separation and purification, catalysis, and sensing^15^. MOFs are usually synthesized through the self-linkage and coordination of metallic species with organic ligands in a cyclic frame with porosity^16,17^.

MIL_Al_-101 is a representative of the broad family of MOFs, which is characterized by high porosity, stability, easy preparation, low cost, and surface functionality, making it an ideal choice for adsorption of contaminants^18^. Many researchers have proved the excellent performance of MILs toward the adsorptive removal of ibuprofen^19^, toxic dyes^20^, sulfacetamide^21^ and ciprofloxacin^22^. Although MOFs-based adsorbents provide some advantages, however, their tendency to aggregate and lose during the recovery process is considered a respective drawback for their applications^23^. Chitosan (Cs) is a natural, non-toxic, and inexpensive polysaccharide that with its unique physical and chemical properties including biocompatibility, biodegradability, flexibility, and various functional groups^24^, has been proposed as a substrate to prevent MOF agglomeration/aggregation in the present study. Furthermore, the presence of free hydroxyl and amino groups in chitosan structure and permission of their chemical modification can facilitate the adsorption of various types of pollutants in chitosan-derived nanocomposites^25^. Therefore, it is expected that the combination of extraordinary properties of chitosan and MIL_Al_-101 for the synthesis of MIL/Cs NCs as a novel adsorbent will have an acceptable performance toward AZT removal. After synthesis of MIL/Cs NCs and their utilization in the removal of AZT from samples, separation and reuse of the adsorbent is necessary and inevitable to reduce the costs of the adsorption process. This process is generally performed with filtration and centrifugation which require a lot of time, cost, and manpower. Hence, induction of magnetic properties to the MIL/Cs NCs using Fe_3_O_4_ NPs and its separation using an external magnet was considered to solve this problem^26,27^.

Given the issues mentioned above, the purpose of present research can be summarized as follows: (a) Synthesis of novel MIL/Cs@Fe_3_O_4_ NCs and its characterization by SEM, TEM, XRD, FTIR, BET and VSM techniques, (b) Modeling and optimization of the AZT adsorption using response surface methodology based central composite design (RSM-CCD), (c) study of isotherms, kinetics and thermodynamics of process, (d) Investigation of the interfering anion presence on the performance of adsorption process, (e) Determination of MIL/Cs@Fe_3_O_4_ NCs desorption and their stability and, (f) Comparison of synthesized adsorbent ability to remove AZT in real conditions with laboratory conditions.

## Materials and methods

### Materials and equipment

Reagents and instruments used in this study were given in Supplementary Information, Text 1.

### Preparation and characterization of adsorbents

#### Chitosan obtained from shrimp shell (Cs)

The chitosan (Cs) synthesis procedure in detail is provided on Supplementary Information, Text 2.

#### NH_2_-MIL_Al_-101

NH_2_-MIL_Al_-101 was synthesized by a microwave method in accordance with a previously reported procedure with some modifications^28^. In detail, 0.51 g of AlCl_3_·6H_2_O and 0.56 g of 2-aminoterephthalic acid were dissolved in 70 mL of DMF. After 30 min stirring, the mixture was placed in a Teflon^®^-lined autoclave and heated at 140 °C by microwave for 3 h. Afterwards the autoclave was allowed to naturally cool to room temperature. The resulting solid product was filtered and washed with acetone for several times, activated with methanol under reflux over-night and evacuated at 200 °C for 6 h.

#### NH_2_-MIL_Al_-101/chitosan (MIL/Cs NCs)

The procedure for the synthesis of NH_2_- MILAl-101/chitosan as described by Wang et al.^18^, was applied with slight modifications. 0.3 g chitosan powders, 1.1 g 2-aminoterephthalic acid, and 0.89 g AlCl_3_·6H_2_O were mixed well and dispersed in 100 mL DMF. The mixture was stirred for 45 min before being introduced into a Teflon autoclave, followed the same procedure as that for synthesis of NH_2_-MIL_Al_-101.

#### NH_2_-MIL_Al_-101/chitosan@Fe_3_O_4_ (MIL/Cs@Fe_3_O_4_ NCs)

MIL/Cs@Fe_3_O_4_ NCs was prepared by co-precipitation under alkaline conditions following our previous published method^29^. 0. 5 g of MIL/Cs NCs was added in 400 mL of iron salt solution consisting of iron (II) chloride (0.03 M) and iron (III) chloride (0.06 M). The obtained mixture was homogenized using an US probe for 30 min (20 kHz, 585 W). Under US irradiation, 30 mL of NaOH solution added drop wise into the mixture until pH kept at 10–11. The suspension was mixed and heated at 80 °C for 1 h followed by cooling. Finally, the obtained black solid was magnetically collected and washed several times with distilled water until pH became neutral. MIL/Cs@Fe_3_O_4_ NCs was dried overnight at 105 °C in a hot air oven and stored in an airtight container for further experiments.

### Characterization

Characteristic analyzes for the synthesized adsorbent are presented in Supplementary Information, Text 3.

### Adsorption procedures

In this study, all the experiments were performed in a 250 mL conical flask containing 100 mL of aqueous solution. To determine the effect of key parameters on the removal efficiency, the sample solutions were agitated in an air shaker at 150 rpm at room temperature. A Central Composite Design (Four factorial CCD) approach in the series of batch tests were developed to optimize the critical parameters in adsorption process (an experimental matrix of the RSM-CCD is listed in Table 1). All experiments were performed in triplicate. The adsorption efficiency (R, %), and capacity (qe, mg/g), were calculated by the following equations:

1 $$\mathrm{R}=\frac{\left({\mathrm{C}}_{0}-{\mathrm{C}}_{\mathrm{e}}\right)}{{\mathrm{C}}_{0}}, $$

2 $$\mathrm{qe }=\frac{\left({\mathrm{C}}_{0}-{\mathrm{C}}_{\mathrm{e}}\right)}{\mathrm{m}} \times \mathrm{V}, $$

where C_0_ (mg/L) and C_e_ (mg/L) are the initial and equilibrium AZT concentrations, respectively; V (L) is the volume of solution, and m (g) is the mass of adsorbent. After optimizing the above-mentioned parameters, kinetics (0–80 min), isotherms (10–200 mg/L) and thermodynamic (278–343 °K) of adsorption process were investigated according to a study by Azari et al.^30^. Desorption and economic feasibility of MIL/Cs@Fe_3_O_4_ NCs toward AZT removal were investigated with ultrasound + chemical modifications in various ethanol concentrations. Finally, effect of background electrolytes on adsorption process and adsorbent performance in real samples was investigated and reported.

**Table 1:** CCD matrix ranges and their response for adsorption of AZT by MIL/Cs@Fe_3_O_4_.

Factor	Name	Units	Minimum	Maximum	coded low	Coded high	Mean	Std. Dev	
A	pH		2	10	4	8	6	1.79	
B	Adsorbent dose	g/L	0.1	0.5	0.2	0.4	0.3	0.09	
C	Time	min	10	90	30	70	50	17.89	
D	AZT Conc	mg/L	10	50	20	40	30	8.94	

Std	Run	Factor 1	Factor 2	Factor 3	Factor 4	Response			
		A	B	C	D	Adsorption efficiency	Predicted efficiency	Remain concentration	Adsorption capacity
		*	g/L	min	mg/L	%	%	mg/L	mg/g
14	1	8	0.2	30	40	85.00	84.26	6.00	170.00
3	2	6	0.3	10	30	55.37	56.45	13.39	55.37
4	3	6	0.3	50	30	73.78	72.56	7.87	73.78
7	4	6	0.3	50	50	47.70	48.10	26.15	79.50
18	5	10	0.3	50	30	97.79	98.19	0.66	97.79
19	6	8	0.4	70	20	94.65	94.58	1.07	47.33
17	7	6	0.5	50	30	69.95	70.35	9.02	41.97
11	8	4	0.2	30	20	30.36	29.62	13.93	30.36
9	9	6	0.3	50	30	75.00	72.56	7.50	75.00
2	10	6	0.3	50	30	71.00	72.56	8.70	71.00
20	11	6	0.3	50	30	73.00	72.56	8.10	73.00
16	12	4	0.2	70	20	41.07	41.00	11.79	41.07
21	13	6	0.1	50	30	53.52	53.92	13.94	160.55
8	14	8	0.2	70	40	82.29	82.22	7.08	164.58
12	15	4	0.4	30	40	41.49	40.75	23.40	41.49
1	16	6	0.3	50	30	70.00	72.56	9.00	70.00
10	17	6	0.3	90	30	72.00	71.73	8.40	72.00
13	18	4	0.4	70	40	49.16	49.09	20.34	49.16
5	19	8	0.4	30	20	82.43	81.69	3.51	41.21
6	20	6	0.3	50	10	80.23	80.63	1.98	26.74
15	21	2	0.3	50	30	23.44	23.84	22.97	23.44

### Adsorption modeling by CCD-DF

To enhance the AZT removal efficiency using the smallest number of potential adsorption tests, a response surface methodology (RSM) based central composite design (CCD) technique (MODDE^®^ Pro Software, V.11.0.2) was applied. Table 1 shows the proposed statistical method to investigate the effect of four independent parameters including pH (2–10), adsorbent dose (0.1–0.5 g/L), AZT concentration (10–50 mg/L) and time (10–90 min) on the response factor (adsorption efficiency, %). In this study, a total of 21 experiments were investigated. At a level of 0.05, ANOVA analysis was used to discover which factors had a significant impact on the response variable. The model parameters were described using analysis of variance (ANOVA), correlation coefficient (R^2^) value, F-test value, and contrasting of experimental Vs. model-predicted data. The model's accuracy and applicability were further evaluated using the probability value (Prob > F), relative standard deviation (RSD), pure error, and Q^2^ value. The operational parameters of the AZT adsorption process were optimized using desirability functions (DF). The selected models' process optimization findings were repeated (n = 5) on a laboratory scale (actual circumstances) until the best and most accurate model for AZT adsorption was found.

## Results and discussion

### Adsorbent characteristics

**Figure 1:**
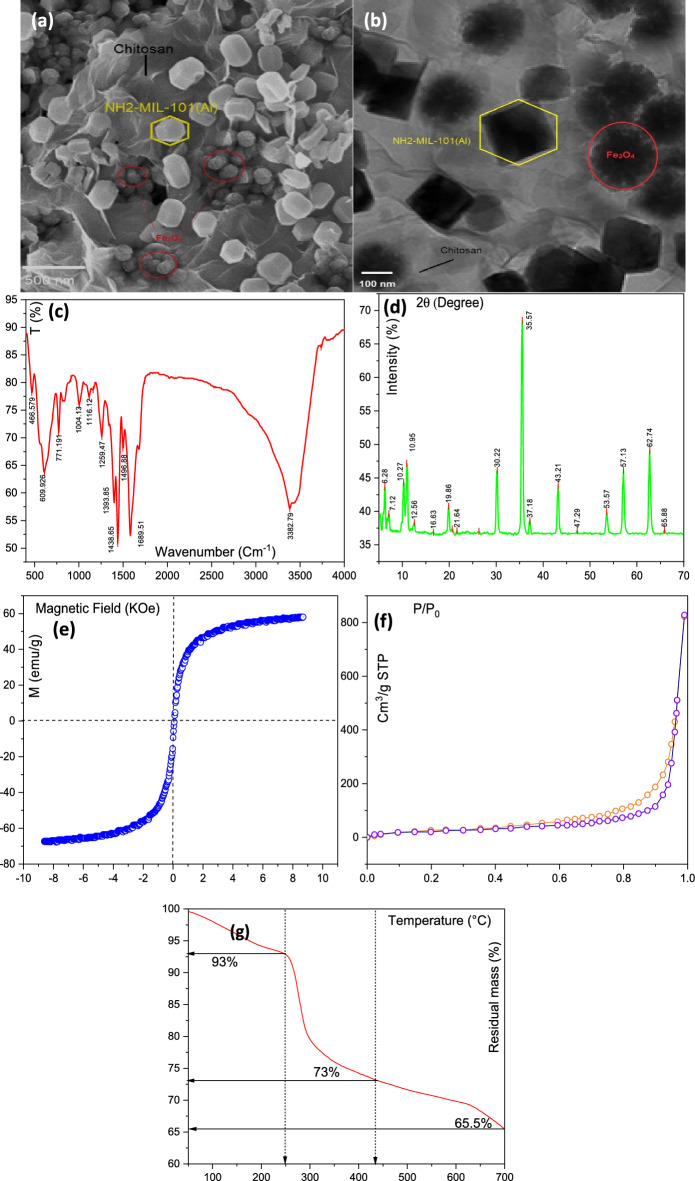
SEM (**a**), TEM (**b**), FTIR (**c**), XRD (**d**), VSM (**e**), BET (**f**) and TGA (**g**) analysis of MIL/Cs@Fe_3_O_4_ NCs.

The SEM analysis of the MIL/Cs@Fe_3_O_4_ NCs is shown in Fig. 1a. As shown, NH_2_-MIL_Al_-101 with hexagonal shape and average diameter of 88.5 ± 10.1 nm are distributed non-uniformly on the surface of chitosan. The surface of chitosan is observed as smooth, sheet-like and layered form. In other sides, Fe_3_O_4_ NPs with spherical structure (23.3 ± 4.0 nm) are observable on the chitosan surface. The image reveals that the surface of the adsorbent is rough and has a good porosity. This feature leads to a better contact of the adsorbent with the pollutant, which resulted in an improvement in adsorption performance. Furthermore, no agglomeration is seen in the MIL/Cs@Fe_3_O_4_ NCs structure which can be related to the presence of chitosan in the structure of the synthesized sample. A TEM analysis was applied to investigate the shape and particle sizes of MIL/Cs@Fe_3_O_4_ NCs, and its results are presented in Fig. 1b. The images indicate that the NH_2_-MIL_Al_-101 and Fe_3_O_4_ nanoparticles are hexagonal and spherical respectively and distributed heterogeneously in a size range of 80–100 nm and 20–40 nm on the chitosan surface, which coincides with results obtained from SEM image. Due to the higher density of NH_2_-MIL_Al_-101 and Fe_3_O_4_ than chitosan, these nanoparticles are recognized in a darker color (black), while chitosan has a light and transparent structure. DLS technique was employed to determine the particle size distribution (Figure is not shown): the obtained average sizes of Fe_3_O_4_ NPs, NH_2_-MIL_Al_-101 and MIL/Cs@Fe_3_O_4_ NCs were 21.75 nm, 85.34 nm and 108.96 nm, respectively. The FTIR spectrum of the adsorbents at a wavelength within 400–4000 cm^−1^ is shown in Fig. 1c. The peak at 609 cm^−1^ is attributed to the Fe–O vibration of Fe_3_O_4_ NPs^31^. The medium peak at 466 cm^−1^ is due to Al–O vibrations, which confirms the formation of the organometallic structure. The broad peak from 3382 cm^−1^ is attributed to the –NH_2_ and –OH groups and the peaks at 1689 and 1598 cm^−1^ are related to the CONH_2_ and NH_2_ groups in the chitosan structure, respectively^32^. The peaks at 1438 and 1496 cm^−1^ were assigned to symmetric and asymmetric stretching of carboxyl groups, respectively. The peak at 1393 and 771 cm^−1^ correspond to the –COO– bond and C–H vibration of the aromatic cycles in the phenyl ring. These bonds at 1438, 1496, 1393 and 771 cm^−1^ suggest the presence of the organic ligands of 2-aminoterephthalic acid. Two peaks appearing at 1259 and 1116 cm^−1^ can be attributed to the stretching vibrations of aromatic functional epoxy C–O and alkoxy C–O groups, respectively. XRD pattern of synthesised MIL/Cs@Fe_3_O_4_ NCs in the range of 2θ = 5–70° is represented in Fig. 1d. The characteristic peaks of NH_2_-MIL_Al_-101 are appeared at 2θ = 6.28°, 7.12°, 8.89°, 10.27° and 17°, which were relatively similar to the patterns of previous references^33,34^. The diffraction peaks at 10.96°, 19.86° and 21.64° are assigned to crystal forms of chitosan. On the other hand, the obtained peaks at 26.3°, 30.22°, 35.57°, 43.21°, 53.57°, 62.74°, and 65.88° related to the diffraction planes of 172°, 220°, 342°, 400°, 511°, 122°, and 106°, respectively confirm the presence of Fe_3_O_4_ crystals in the MIL/Cs@Fe_3_O_4_ NCs structure (0866-088-01 JCPDS NO.). The above results indicate that NH_2_-MIL_Al_-101 and Fe_3_O_4_ NPs are loaded onto the CS and MIL/Cs@Fe_3_O_4_ NCs was synthesized successfully. Magnetic measurements and separation of MIL/Cs@Fe_3_O_4_ NCs were taken with a VSM analysis at room temperature, as presented in Fig. 1e. This analysis was performed in a magnetic field of ± 10 KOe and saturation magnetization around ± 80 emu/g. The results showed that the saturation magnetization (Ms) of MIL/Cs@Fe_3_O_4_ NCs was observed around 60.77 emu/g. The result clearly demonstrated the appropriate magnetic property of the synthesized adsorbent. The Nitrogen adsorption–desorption isotherms and the pore size distribution of the MIL/Cs@Fe_3_O_4_ NCs is shown in Fig. 1f and Supplementary Table S1. The isotherms are identified as type IV with H_4_ typical hysteresis loops, which are the characteristic isotherm of mesoporous materials according to IUPAC classification. The BET surface area and average pore diameters of the of the product were also calculated as 451.73 m^2^/g and 2.78 nm, respectively. The BET surface area calculated for the MIL/Cs@Fe_3_O_4_ NCs is ~ 1.5 times higher than the Fe_3_O_4_@C nanocomposite synthesized by Van Tran et al.^35^. Although these values are higher than all samples (Fe_3_O_4_ NPs, NH_2_-MIL_Al_-101 and MIL/Cs NCs), but this difference between MIL/Cs@Fe_3_O_4_ NCs and MIL/Cs NCs is not too large. This phenomenon may be due to the occupation of the porosity of MIL/Cs NCs by magnetic nanoparticles (Fe_3_O_4_) and/or clotting (agglomeration) of the nanocomposite. Thermal stabilities of MIL/Cs@Fe_3_O_4_ NCs were investigated by using TGA analysis (Fig. 1g). MIL/Cs@Fe_3_O_4_ NCs showed gradual weight loss within three steps. The first step occurred in the temperature range of 50–250 °C with a weight loss of 7%, attributed to the evaporation of solvent or water molecules inside the framework. A weight loss of 20% in the temperature range of 150–440 °C could be ascribed to the decomposition of guest molecules or unreacted products inside the ligand structure. In the final step, beyond 440 °C, MIL/Cs@Fe_3_O_4_ NCs structure degraded. The thermal stability results revealed the good stability of the synthesized adsorbent.

### AZT removal under different systems

**Figure 2:**
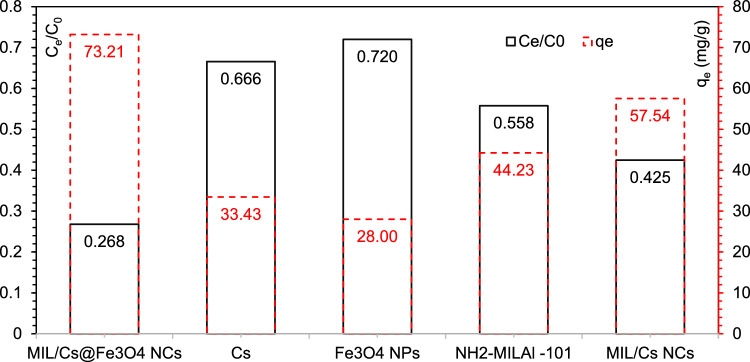
Comparison of AZT removal with different adsorbents under same conditions.

Figure 2 shows the ability of MIL/Cs@Fe_3_O_4_ NCs compared to Cs, Fe_3_O_4_, NH_2_-MIL_Al_-101 and MIL/Cs NCs for AZT removal under similar experimental conditions (experiment conditions: Contact time: 60 min, pH: 7 ± 0.4, Sample dose: 0.5 g/L, AZT Conc.: 50 mg/L). AZT removal efficiencies by Cs and Fe_3_O_4_ were negligible (33.43 and 28%, respectively), showing that AZT cannot be effectively removed by Cs and Fe_3_O_4_. However, higher removal efficiencies were observed when Cs and Fe_3_O_4_ were coupled with NH_2_-MIL_Al_-101 i.e., MIL/Cs NCs and MIL/Cs@Fe_3_O_4_ NCs. The removal efficiencies of AZT by NH_2_-MIL_Al_-101, MIL/Cs NCs and MIL/Cs@Fe_3_O_4_ NCs were 44.23, 57.54 and 73.21% within 60 min, respectively. Higher surface area, better average pore volume/ size and more functional groups seem to be the reasons for the superior performance of MIL/Cs@Fe_3_O_4_ NCs over other adsorbents. The priority and order of samples in AZT removal were obtained as MIL/Cs@Fe_3_O_4_ NCs > MIL/Cs NCs > NH_2_-MIL_Al_-101 > Cs > Fe_3_O_4_, respectively. Accordingly, MIL/Cs@Fe_3_O_4_ NCs was selected as the preferred adsorbent in AZT removal and was used in following experiments.

### Effect of key parameters on adsorption of AZT

**Figure 3:**
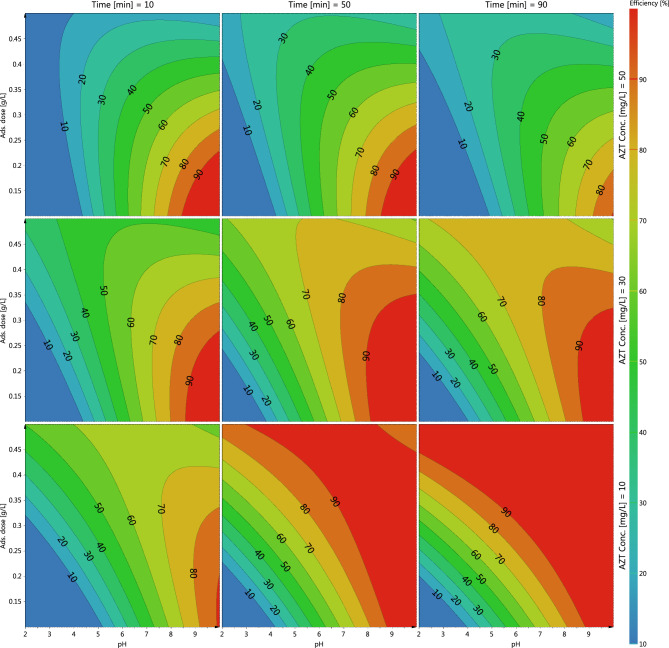
4D plots of variables effect on AZT removal.

The effect of pH changes in the range of 2 to 10 on AZT adsorption by MIL/Cs@Fe_3_O_4_ NCs was investigated. As can be seen from Fig. 3, the removal efficiency is improved by increasing the pH so that the highest efficiency is observed in alkaline conditions and the lowest was obtained at pH < 5. In other side, the point of zero charge (pH_pzc_) values for MIL/Cs@Fe_3_O_4_ NCs was obtained to be 4.6 (Supplementary Fig. S1). The increasing trend of AZT removal with increasing the pH of the solution can be associated with the fact that pHpzc (4.6) of adsorbent is lower than the pKa (8.7) of AZT (pH_pzc_ < pH < pKa). Therefore, at pH =  ~ 8, the surface charges of the adsorbent and the adsorbate become opposites charge and an electrostatic attraction occurs between them which results in maximum adsorption. As a result, the optimal pH 8 was employed in further adsorption tests. Imani poor et al. (2021) studied the adsorption of azithromycin onto L-methionine modified montmorillonite K10 and 3-aminopropyltriethoxysilane functionalized magnesium phyllosilicate organoclays. They found that the maximum adsorption capacity obtained at a pH 8.0 ± 0.1^36^. A similar result was observed for azithromycin removal using powdered zeolites in research by Sousa et al.^37^. They reported that the lowest efficiency was recorded under acidic pH (2.5–4.5), while alkaline conditions improved the performance of the azithromycin adsorption process. The effects of adsorbent dosage (from 0.1 to 0.5 g/L) on adsorption of AZT by MIL/Cs@Fe_3_O_4_ NCs was investigated at pH = 8.0, contact time = 60 min, and room temperature. Based on the results, the increase in the adsorbent dose has led to an increase in adsorption efficiency from 53 to 76%. The specific surface area and large quantity of reactive surface centers or functional groups of the adsorbent towards contaminates might be associated to adsorption percentage improvement as adsorbent increases^38,39^. Wahab et al. found that increasing the amount of magnetic activated carbon (MAC) from 0.01 to 0.15 g can increase the removal efficiency of AZT from ~ 10 to 100%^40^. In azithromycin adsorption onto modified natural clinoptilolite, Saadi et al. observed a direct relationship between process effectiveness and adsorbent dose. They reported that the initial quick adsorption was attributed to the unoccupied site and surface area of absorbent, resulting in successful adsorbate diffusion within the adsorbent^41^. The adsorption of AZT on MIL/Cs@Fe_3_O_4_ NCs was studied by adjusting contact time in the optimized pH and adsorbent dosage. From Fig. 3, increasing the contact time from 10 to 90 min has resulted in a 20% improvement in efficiency. Efficiency improvements with increasing time can be due to increase the contact and collisions of AZT with the MIL/Cs@Fe_3_O_4_ NCs, which subsequently improves the mass transfer of contaminates on to the adsorbent. Adsorbents generally have a limited and specific adsorption capacity and become saturated after reaching equilibrium time. The proper efficiency at the beginning of the adsorption process can be linked to the existence of large unoccupied sites and functional groups on the adsorbents that begin to fill over time^42^. A more detailed about the effect of contact time on azithromycin adsorption has been discussed and reported in the kinetics study section. Balarak et al. investigated the effect of contact time on AZT molecule adsorption on Azolla Filiculoides-based activated porous carbon ranging from 0 to 150 min. They found that increasing the contact time leads to an increase in percentage removal^43^. Other researchers have observed similar results in the adsorption of different antibiotics by modified alpha alumina nanoparticles and NaY zeolite synthesized from wheat straws ash^44,45^. Increasing the initial concentrations from 10 to 50 mg/L has resulted in a considerable drop in efficiency, as seen in Fig. 3. The inverse relationship between AZT concentration and efficiency is owed to the saturation of accessible active sites on MIL/Cs@Fe_3_O_4_ NCs. On the contrary, increasing the initial concentration of AZT has led to an increase in adsorbate per mass of adsorbent (qe, mg/g). The increased in qe (mg/g) at higher concentrations might be explained by the significant driving force for mass exchange at a large initial concentration^46^. Davoodi et al. studied the removal of AZT using raw and saponin-modified nano diatomite by varying the initial AZM concentration from 20 to 100 mg/L. They noticed that increasing the pollutant concentration causes a significant reduction in the adsorption process^47^. In 2020, Ardakani et al. used from PECVD film to evaluate the influence of initial concentration on AZT removal^48^. According to the Ardakani report’s, increasing the AZT concentration from 0 to 160 mg/L has resulted in a decrease in efficiency, and the highest efficiency has been obtained in the lowest concentration. Rapid saturation of active sites with increasing pollutant concentration was the main cause of efficiency reduction.

### Statistical analysis and modeling of AZT adsorption

The validity of the model and operational parameters was investigated by RSM-CCD technique and ANOVA analysis. Table 2 summarizes the ANOVA results for the selected model. The developed model can well describe and predicted the adsorption of AZT by MIL/Cs@Fe_3_O_4_ NCs, as evidenced by the high values of R^2^ (> 0.997), Adj R^2^ (> 0.991), and Model F-value (170.46). Furthermore, the R^2^ value has reasonably agreed with the Adjusted R^2^ value; i.e., the difference is lesser than 0.2. On the other hand, RSD (1.873) confirms the model's suitability for interpreting the studied process. The signal-to-noise ratio is expressed by the adequate precision (A–P) parameter. This value was found to be 46.973 (A–P > 4) which indicates an adequate signal and model could be used to navigate the design (good match between experimental and computed results)^49^. The p-value of the lack of fit parameter was 6.2431e−01 (greater than 0.05), indicating that the developed model was appropriate and the predicted values are accurate^50^. Variance analysis was also used to investigate the effects of variables. The results show that all parameters had a considerable influence on the AZT adsorption except interaction between pH and time, pH and AZT concentration and adsorbent dose and time. The equation in terms of actual factors can be used to make predictions about the response for given levels of each factor. The equation of AZT adsorption on MIL/Cs@Fe_3_O_4_ NCs can be defined as follows:

**Table 2:** Analysis of variance (ANOVA) for CCD modeling and process optimization results.

Model statistics						
Removal	DF	SS	MS	F-value	p-value	SD
Total	21	9.7665e + 04	4.6507e + 03			
Constant	1	8.9274e + 04	8.9274e + 04			
Total corrected	20	8.3912e + 03	4.1956e + 02			2.0483e + 01
Model	14	8.3702e + 03	5.9787e + 02	1.7046e + 02	**1.3101e−06**	2.4451e + 01
Residual	6	2.1044e + 01	3.5074e + 00			1.8728e + 00
Lack of Fit	2	4.4166e + 00	2.2083e + 00	5.3122e−01	**6.2431e−01**	1.4860e + 00
Pure error	4	1.6628e + 01	4.1570e + 00			2.0389e + 00
Q^2^	0.949			A–P	46.9730	
R^2^	0.997			RSD	1.873	
R^2^ adj	0.992			Predicted R^2^	0.9486	

Parameters statistics						
	SS	MS	F-value	p-value	Effect	
pH	2.7639E + 03	2.7639E + 03	7.8802E + 02	< 0.0001	74.349	
Ads. dose	1.3494E + 02	1.3494E + 02	3.8470E + 01	8.0000E−04	16.4282	
Time	2.3371E + 02	2.3371E + 02	6.6630E + 01	2.0000E−04	15.2875	
AZT Conc	5.2904E + 02	5.2904E + 02	1.5084E + 02	< 0.0001	− 32.5281	
pH*Ads. dose	3.4674E + 02	3.4674E + 02	9.8860E + 01	< 0.0001	− 74.4833	
pH*Time	9.8000E + 00	9.8000E + 00	2.7900E + 00	1.4570E−01	− 8.8535	
pH*AZT Conc	9.2730E−01	9.2730E−01	2.6440E−01	6.2550E−01	3.85191	
Ads. dose*Time	1.7700E + 01	1.7700E + 01	5.0500E + 00	6.5800E−02	11.9001	
Ads. dose*AZT Conc	7.0550E + 01	7.0550E + 01	2.0120E + 01	4.2000E−03	− 33.5981	
Time*AZT Conc	4.0360E + 01	4.0360E + 01	1.1510E + 01	1.4600E−02	− 17.9681	
pH ^2^	2.0894E + 02	2.0894E + 02	5.9570E + 01	2.0000E−04	− 23.0822	
Ads. dose ^2^	1.7034E + 02	1.7034E + 02	4.8560E + 01	4.0000E−04	− 20.8412	
Time ^2^	1.1244E + 02	1.1244E + 02	3.2060E + 01	1.3000E−03	− 16.9331	
AZT Conc.^2^	1.0520E + 02	1.0520E + 02	2.9990E + 01	1.5000E−03	− 16.3786	

Optimization process						
Factor	Value	Unit	RSD	Contribution	Failure	DF
pH	7.992	*	–	64.0637	4%	0.996
Ads. dose	0.279	g/L		20.1934		
Time	64.256	min	–	1.20639		
AZT Conc	10.107	mg/L	–	14.5365		
Efficiency	98.362	%	± 3.24	100		
Repeat (n = 5)	96.021	%	± 4.23	*		

3 $$\mathrm{Efficiency }(\mathrm{\%}) = 72.56 + 18.59(\mathrm{pH}) + 4.11(\mathrm{Ads}.\mathrm{ dose}) +3.82 (\mathrm{Time}) -16.26 (\mathrm{AZT Conc}.) -9.31 (\mathrm{pH}\times \mathrm{Ads}.\mathrm{ dose}) -1.11(\mathrm{pH}\times \mathrm{Time}) +0.963(\mathrm{pH}\times \mathrm{ AZT Conc}.) +1.49 (\mathrm{Ads}.\mathrm{ dose}\times \mathrm{ Time}) -8.40 (\mathrm{Ads}.\mathrm{ dose}\times \mathrm{ AZT Conc}.) -4.49 (\mathrm{Time}\times \mathrm{ AZT Conc}.) -2.89 {(\mathrm{pH})}^{2} -2.61{(\mathrm{Ads}.\mathrm{ dose})} -2.12 {(\mathrm{Time})}-8.19 {(\mathrm{AZT Conc}.)}^{2}$$

It can be seen that the $\mathrm{pH}$, $\mathrm{Ads}.\mathrm{ dose}$**,** $\mathrm{Time}$, $\mathrm{pH}\times \mathrm{ AZT Conc}.$ and $\mathrm{Ads}.\mathrm{ dose}\times \mathrm{ Time}$ parameters have positive signs, and the others are negative. These findings show that increasing the mentioned factors has a synergistic effect on the adsorption process; nevertheless, increasing other parameters reduces the efficiency. According to Eq. (3) and Table 2, pH with the highest coefficient has the greatest impact on the AZT adsorption process by MIL/Cs@Fe_3_O_4_ NCs. Plotting actual data Vs. predicted data with R^2^ > 0.948 illustrated that the developed model has a strong ability to predict removal efficiency (Supplementary Fig. S2a). The normal probability plot of the studentized residuals (Supplementary Fig. S2b), shows that the plotted points are close to a straight line (balanced and uniform distribution of residuals around the normalcy line.) that indicates the model has been well fitted with the experimental results. Supplementary Fig. S2c shows the residuals are randomly scattered near the horizontal zero reference (± 3) indicating a good fit of the model for AZT adsorption by MIL/Cs@Fe_3_O_4_ NCs.

### Process optimization

After determination of the effect of each parameter (positive and negative roles) on the adsorption process, calculating the optimum conditions is essential and inevitable^51^. The purpose of optimization was improvement of AZT adsorption efficiency in the batch process, so the response parameter (AZT adsorption efficiency) was set to maximum level to determine the best performance of system. The lower and upper limit values of other variables were taken from the experimental data ranges (see Table 1). Based on the DF approach results, the maximum adsorption efficiency for AZT was 98.362 ± 3.24% at pH = 7.992, AZT conc. = 10.107 mg/L, ads. dose g/L = 0.279 and time equal to 64.256 min. To determine the accuracy of the software model results, optimized conditions (the above result) were simulated in laboratory scale and repeated 5 times. The evidence suggested that the simulated test results (96.021 ± 4.23%) are consistent with the model's results (98.362 ± 3.24%), so the result recorded by DF was used were used in the isotherms, kinetics and thermodynamic investigation. The percentage of parameters participation in maximizing the AZT adsorption process also revealed that pH and time with 64.063 and 1.206%, had the highest and lowest impact on system optimization, respectively.

### Adsorption isotherms

To determine the best-fitting isotherm model with experimental data, the Langmuir, Freundlich, Temkin, and Dobbin-Radoshkvich models were studied (conditions: pH = 7.992, AZT Conc. = 10.107 mg/L, Ads. dose g/L = 0.279). From Table 3 and Supplementary data, Fig. S3, The R^2^ value for the Langmuir isotherm (R^2^ > 0.9981) is higher than other models (0.9759, 0.9396, and 0.7093 for Freundlich, Temkin, and Dobbin-Radoshkvich, respectively), indicating that experimental data is in well agreement with Langmuir model. The lower value of χ^2^ error (0.011) also confirms the results obtained. According to the Langmuir model, the AZT is adsorbed in a monolayer on a homogeneous surface of adsorbent with uniform energies for all the binding sites without any interaction between the molecules^52,53^. The adsorption capacity upon the Langmuir model was obtained as 238.5527 mg of AZT per gram of MIL/Cs@Fe_3_O_4_ NCs. The dimensionless separation factor (RL) of the Langmuir adsorption isotherm was used to determine the favourability of the adsorption process (irreversible → RL = 0, favourable → 0 < RL < 1, linear → RL = 1 and unfavourable → RL > 1). All the RL values lie in the range of 0 < RL < 1, which suggests that the AZT are favourably adsorbed on MIL/Cs@Fe_3_O_4_ NCs. Compared to Langmuir isotherm model, Freundlich isotherm model had the poorest fit with experimental data, as determined by comparison of R^2^ and χ^2^ values. However, high values of k_F_ (60.837 L mg^-1^) and n parameter (greater than 1) indicated that adsorption of adsorbate on adsorbent was acceptable/feasible^54,55^. Table 3 reveals that the experimental data are less consistent with the Temkin isotherm model than either of the previous two models. Nevertheless, the positivity of Temkin isotherm constants (B = 32.042) proves that the adsorption process in the present study is endothermic in nature. The results indicated that experimental data fitted with the D–R isotherm with R^2^ > 0.709 under the optimized condition. Magnitude of adsorption energy, E, for AZT adsorption on the MIL/Cs@Fe_3_O_4_ NCs adsorbent was calculated to be 2704.0065 kJ/mol (> 40 kJ/mol), representing that the adsorption is chemisorption process^56,57^. Non-linear results of the studied isotherms (Fig. S3e) also clearly showed compliance of the experimental data with the Langmuir isotherm.

**Table 3:** Isotherm, kinetic and thermodynamic parameters for adsorption of AZT onto MIL/Cs@Fe_3_O_4_ NCs NCS adsorbent.

Isotherms					
Model	Equation	Nomenclature	Parameters	Values	X^2^
Langmuir	C_e_/q_e_ = C_e_/Q_m_ + 1/K_a_Q_m_	The slope and intercept of the linear plot of C_e_/q_e_ versus C_e_ give Q_m_ and K_a_, respectively	Q_m_ (mg g^−1^)	238.5527	0.011
			K_L_ (L mg^−1^)	0.19614	
			R_L_	0.1812	
			R^2^	0.9981	
Freundlich	Ln q_e_ = (1/n) ln C_e_ + lnK_F_	The slope and intercept of the linear plot of lnq_e_ versus ln C_e_ give 1/n and K_F_, respectively	n	3.3661	0.024
			K_F_ (L mg^−1^)	60.837	
			R^2^	0.9759	
Temkin	q_e_ = B_l_ ln C_e_ + B_l_ ln K_T_	B_1_ and K_T_ are calculated from the slope and intercept of the linear plot of q_e_ against lnC_e_, respectively	B_l_	32.0417	0.031
			K_T_ (L mg^−1^)	10.04241	
			R^2^	0.9396	
Dubinin and Radushkevich	Ln q_e_ = − Kε^2^ + ln Q_s_, (ε = RT ln (1 + 1/C_e_))	The slope and intercept of the linear plot of lnq_e_ versus ε^2^ give K and Q_s_, respectively	E (kJ mol^–1^)	2704.0065	0.046
			D (mol^–2^ kJ^–2^)	6.8384E−08	
			R^2^	0.7093	

Kinetics					
Model	Equation	Nomenclature	Parameters	Values	X^2^
Pseudo-first-order	Ln (q_e_ − q_t_) = − k_1_t + ln(q_e_)	The slope and intercept of the linear plot of ln (q_e_–q_t_) versus t give k_1_ and q_e_, respectively	k_1_ (min^−1^)	0.000157474	0.032
			q_e_ (mg/g)	82.49361895	
			R^2^	0.9833	
Pseudo-second-order	t/q_t_ = t/q_e_ + 1/(k_2_q_e_)^2^	The slope and intercept of the linear plot of t/q_t_ versus t give q_e_ and k_2_, respectively	k_2_ (g/mg^−1^ min^−1^)	0.035565425	0.013
			q_e_ (mg/g)	37.6004392	
			R^2^	0.9991	
Intraparticle diffusion	q_t_ = K_diff_ t^1/2^ + C	The slope and intercept of the linear plot of q_t_ versus t^1/2^ give K_diff_ and C, respectively	K_dif_ (g/mg^−1^ min-0.5)	7.988476742	0.024
			C	5.621384922	
			R^2^	0.9979	
Elovich	q_t_ = 1/β ln(t) + 1/β ln(αβ)	β and α are obtained from the slope and intercept of the plot of q_t_ versus ln (t), respectively	β (g mg^−1^)	9.950388641	0.039
			α (g mg^−1^ min^−1^)	0.029242544	
			R^2^	0.9543	

Thermodynamic					
Temperature	ln k_d_	∆S° (kJ/mol K)	∆G° (kJ/mol)	∆H° (kJ/mol)	qe
278 °K	3.5	0.22	− 8.08	755.23	32.68
288 °K	4.15		− 9.94		34.29
298 °K	5.37		− 13.31		35.63
328 °K	6.58		− 17.95		36.05
343 °K	8.01		− 22.85		36.18

### Adsorption kinetics

As can be seen from the Supplementary data, Fig. S4e, the adsorption efficiency increased sharply at the initial 60 min of the experiment, whereas negligible changes were observed thereafter, which indicates that the adsorption process has reached equilibrium state. The high adsorption rate at the beginning of process may be explained by the high affinity of MIL/Cs@Fe_3_O_4_ NCs for trapping pollutants due to the huge number of vacant sites (active sites) on adsorbent. Over time, the active sites in the adsorbent structure are occupied (filled) by pollutant molecules, which results in reduced efficiency and rate of AZT adsorption^58,59^. In the following, four conventional kinetic models including pseudo-first-order, pseudo-second-order, Elovich, and intraparticle diffusion, were employed to investigate the kinetics of AZT adsorption on MIL/Cs@Fe_3_O_4_ NCs under optimized conditions (see Supplementary data, Fig. S4a–d). Furthermore, the parameters related to the kinetic models i.e., error functions, and regression correlation coefficients (R^2^), are listed in Table 3. A pseudo-first-order model was found to be in good agreement with experimental data with a high R^2^ > 0.983, and χ^2^ values less than 0.032. Higher R^2^ values (0.9991) and lower χ^2^ rate (0.013) on the one hand and more compatibility between experimental qe (qe,exp: 35.86 mg/g) with the calculated qe (qe,cal: 37.60 mg/g) on the other hand clearly indicated that the pseudo-second-order model better describe the adsorption behaviour of AZT onto MIL/Cs@Fe_3_O_4_ NCs. The error values (χ^2^ = 0.039) and R^2^ parameter (0.9543) obtained from the Elovich model indicate that there is the least agreement between the experimental data and this model, and Elovich model is not suitable for describing the experimental data compared to the previous two models. The results obtained so far confirmed that the dominant mechanism of adsorption process is chemisorption^60^. The results also depicted that intraparticle diffusion model with R^2^ > 0.997 and χ^2^ = 0.024 has a higher ability than pseudo-first-order and Elovich models in interpreting data, while it has a weaker performance than pseudo-second-order kinetic model. Besides, the resulting fitting line in the intraparticle diffusion plot (q_t_ against t^0.5^) did not pass through the origin. This shows that intraparticle diffusion was a rate-controlling step (rate-limiting mechanism) in the AZT adsorption process, along with the chemisorption reaction^61,62^.

### Adsorption thermodynamic

Supplementary data, Fig. S5a shows the effect of different temperatures on the AZT adsorption efficiency at the obtained optimized conditions. As it turns out, with increasing temperature from 278 to 343 °K, adsorption capacities improved from 32.68 to 36.18 mg/g. For evaluating feasibility and nature of AZT adsorption process on MIL/Cs@Fe_3_O_4_ NCs, the thermodynamic parameters i.e., Gibbs free energy (ΔG°), enthalpy change (ΔH°), and entropy change (ΔS°) were investigated ant their results are given in Table 3. The quantities of enthalpy and entropy were calculated from slope and intercept of the plot of ln ln K_d_ vs. 1/T (Van't Hoff plot, Supplementary data, Fig. S5b). The values of ΔH° and ΔS° were found to be 755.23 and 0.22 kJ/mol, respectively. Positive value of ΔH° and also the directed trend between ln K_d_ and temperature implied that AZT adsorption on the MIL/Cs@Fe_3_O_4_ NCs was endothermic in nature, indicating that higher temperature enhanced adsorption capacity. ΔS° value became positive, so the increased randomness can be expected at the solid/solution interface during AZT adsorption. Besides, positive ΔS° value corresponded to an increase in the freedom degree of the adsorbed species^63^. Negative values of ΔG° (− 8.08 to − 22.85 kJ/mol) indicated feasibility and spontaneous nature of adsorption process. Moreover, the degree of spontaneity was increased by increasing temperature.

### Effect of background ions

**Figure 4:**
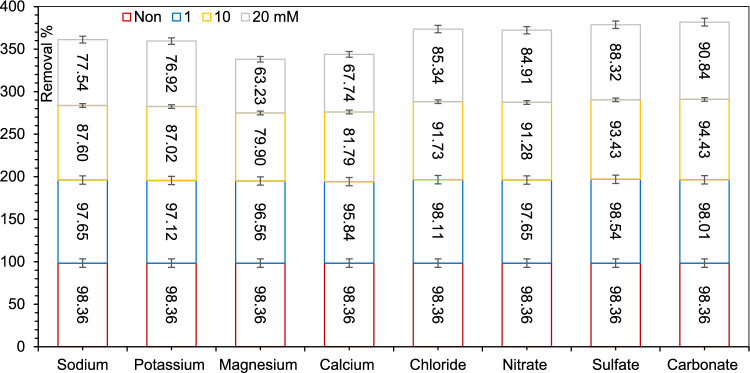
Effect of interfering ions on the efficiency of AZT adsorption under optimized conditions.

The effect of background electrolytes at an initial concentration of 1, 10 and 20 mM on AZT adsorption by MIL/Cs@Fe3O4 NCs are shown Fig. 4. It was found that the Na^+^ and K^+^ cations had a smaller effect on antibiotic adsorption than Mg^2+^ and Ca^2+^ ions. This might be explained by the fact that the high polarization power of divalent ions caused them to exert a stronger squeezing-out effect. The following reasons are also given by the researchers: (a) A dense water hydration shell might surround the adsorbed Mg^2+^ and Ca^2+^ ions could hinder the available adsorption sites by blocking the hydrophobic adsorption region, and (b) It is possible that Mg^2+^ and Ca^2^ compete directly for adsorption sites on MIL/Cs@Fe_3_O_4_ NCs due to inner-sphere complexation, which inhibits the formation of charge-assisted H-bonds with antibiotics. AZT adsorption by MIL/Cs@Fe_3_O_4_ NCs was not significantly affected by background anions, including Cl^−^, NO_3_^−^, SO_4_^2−^ and CO_3_^2−^_,_ which may be due to the strong repellence/repulsion between anions and the negatively charged adsorbent surface^64^.

### Desorption and stability

**Figure 5:**
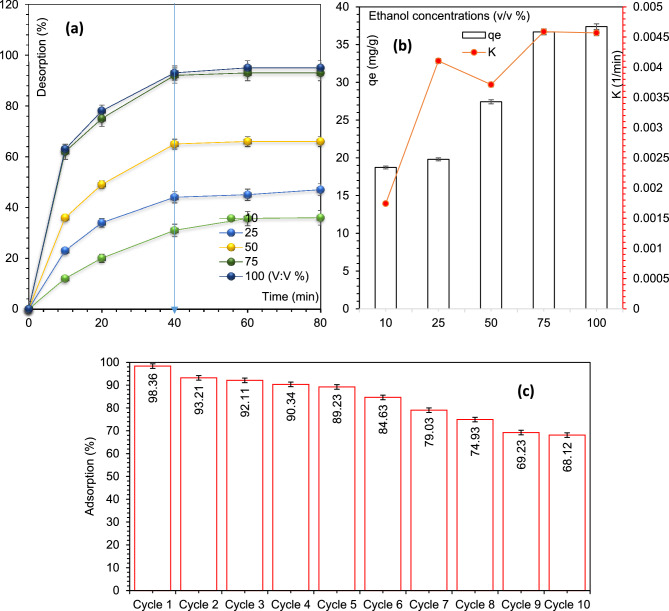
Desorption percentage of AZT at different ethanol concentration (**a**), desorption capacity and the rate of desorption under various ethanol concentrations (**b**) and reusability of MIL/Cs@Fe_3_O_4_ NCs after AZT treatment for 10 cycles (**c**).

To investigate the desorption and economic feasibility of MIL/Cs@Fe_3_O_4_ NCs toward AZT removal, the adsorbent was recycled with ultrasound + chemical modifications in various ethanol concentrations ranging from 10 to 100 (v/v percent). Figure 5a shows the amount of desorption against time at various ethanol concentrations under optimized conditions. As shown, the desorption percentage rose dramatically at various ethanol concentrations and reached to equilibrium in 40 min. In Fig. 5a,b, it can also be seen that the efficiency (%), capacity (mg/g) and rate (k, based on pseudo-second order kinetic) of desorption process have an upward trend up to the ethanol concentrations of 75%, but after that no further improvement has been observed. In the following, the adsorbent's reusability was evaluated for 10 consecutive cycles. For this purpose, after each cycle, MIL/Cs@Fe_3_O_4_ NCs were washed with ethanol and distilled water (75% v/v). As observed from Fig. 5c, the removal efficiency has decreased by only 9.13% during the first to fifth cycles, and this reduction in the adsorption has reached 30.24% in the tenth cycle. The decrease in efficiency can be ascribed to the loss of adsorbent mass and the filling of active sites or functional groups of the adsorbent during repeated runs. The amounts of leached aluminium and iron were 0.024 and 0.147 mg/L in the first cycle, and fell to 0.009 and 0.11 mg/L in the tenth cycle. The leaching of Al and Fe amounts in repeated runs is reasonable (Less than the water standard level), which proves the stability and durability of MIL/Cs@Fe_3_O_4_ NCs for AZT treatment.

### Adsorption mechanism

**Figure 6:**
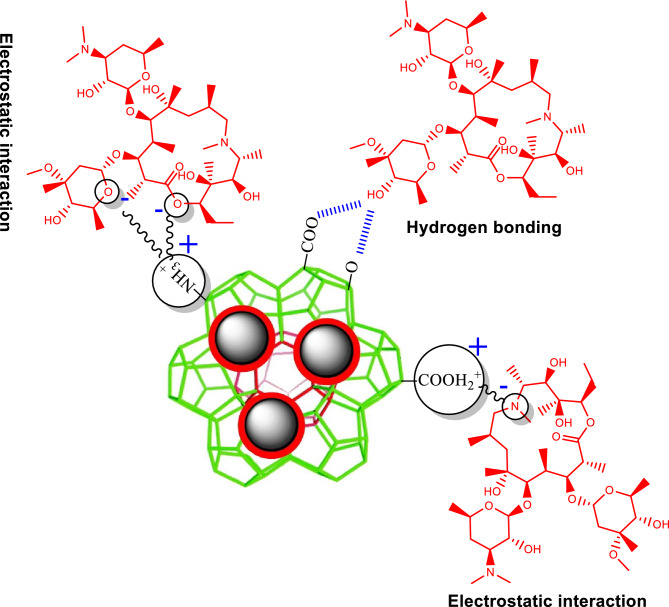
Possible mechanism of AZM adsorption on MIL/Cs@Fe_3_O_4_ NCs.

The mechanism of organic compounds’ adsorption is usually described by hydrogen bonding, electrostatic interaction, and π–π interaction. MIL/Cs@Fe_3_O_4_ NCs used in this study are enriched with π-electrons, amino group (–NH_2_), and acidic carboxyl group (–COOH) that can play an important role in the adsorption mechanism between MIL/Cs@Fe_3_O_4_ NCs and AZM through hydrogen bonding (H-bonds), electrostatic interaction, and π–π interactions. As can be seen in Fig. S6, AZM is surrounded by high amounts of –OH, −CH_3_, =O, –O–, –N–, –N=, and –OCH_3_ functional groups. The aforementioned functional groups can play a role in AZM adsorption as follows: (a) –OH involved to form hydrogen bonding, (b) =O, –O–, –N–, –N=, and –O–CH_3_ groups involve in anionic electrostatic interactions, and (c) –CH_3_ group participates in cationic electrostatic interactions. Figure 6 shows possible adsorption interactions between AZM and MIL/Cs@Fe_3_O_4_ NCs. Since there is no π-electron in AZM, it is unlikely to have π–π interactions with MIL/Cs@Fe_3_O_4_ NCs^65^. Based on the evidence, adsorption may occur as a result of cationic and anionic electrostatic interactions and H-bonding between AZM and MIL/Cs@Fe_3_O_4_ NCs.

### Study of real samples

In order to evaluate the effectiveness and potential of the MIL/Cs@Fe_3_O_4_ NCs for AZT adsorption in real conditions, a series of experiments on tap water, surface runoff, raw wastewater, and secondary effluent were considered under optimized conditions (AZT concentration spiked: 10 mg/L). In Supplementary data, Table S2, the specifications of samples are listed. As can be seen, all samples have lower efficiency compared to DI-water. For example, the efficiency in tap water was 90.45% while the adsorbent performance in surface runoff, raw and treated wastewater were 85.34%, 48.45%, and 65%, respectively (Supplementary data, Fig. S7). The decrease in AZT adsorption efficiency can be attributed to the presence of TDS, various ions, organic compounds, and other contaminants in the actual samples. Based on the results, it can be reported that MIL/Cs@Fe_3_O_4_ NCs has an acceptable ability to remove AZT from aqueous samples under real conditions, although this performance has been significantly reduced in raw wastewater samples.

### Comparison with previous research

The qm parameter of synthesized adsorbent (adsorption capability based on the Langmuir isotherm model, mg/g) was compared with similar literature in terms of adsorbent type, and the results are summarized in Table 4. It was observed that the MIL/Cs@Fe_3_O_4_ NCs adsorbent showed good ability to compete with similar systems for treatment of AZT and has the proper place among the literature. It should be noted that the differences between the adsorption capacities in the various adsorbents can be related to the type of materials used (due to variability in size, surface area, number of active sites, and functional groups), adsorbent dosage and the concentration of the AZT in experiments. Available evidence confirms that the adsorbent synthesized in the present study can have a suitable and promising performance in the removal of AZT as a representative of macrolide antibiotics.

**Table 4:** Comparison of AZT adsorption reported in the literature with the proposed systems.

Adsorbent	C_0_ (mg/L)	Time (min)	Dose (g/L)	q_m_ (mg/g)	Isotherm	Kinetic	References
ZnO/Si	15	45	0.025	213.32	L	PSO	^48^
Raw nano diatomite	100	60	2.5	68	L	–	^47^
Saponin modified nano diatomite	100	3	1	91.7	L	–	^47^
LMP clay	50	90	0.5	298.78	F	PSO	^36^
AMP clay	50	90	0.5	286.10	F	PSO	^36^
FAU-1	10	30	0.1	8.50	F	PSO	^37^
MIL/CS@Fe_3_O_4_ NCS	10.107	64.256	0.279	238.55	L	PSO	This study

*L* Langmuir, *F* Freundlich, *PSO* Pseudo-second-order.

## Conclusions

Magnetic NH_2_-MIL-101(Al)/Chitosan nanocomposite (MIL/Cs@Fe_3_O_4_ NCs) adsorbent was synthesized and developed for AZT treatment from queues solution. RSM-CCD approach was applied to predict and optimize the effect of key factors on AZT removal. This research led to the following results: (1) he results of ANOVA test revealed that the effect of all parameters on the performance of the AZT adsorption was significant with Prob > F <  = 0.05 except pH × Time, pH × AZT concentration and Adsorbent dose × Time, (2) The higher adsorption efficiency (predicted: 98.362%, Real: 96.021) is obtained at pH = 7.992, adsorbent dose = 0.279 g/L, time = 64.256 min and the initial concentration = 10.107 mg/L with DF = 0.996, (3) The isotherms and kinetics of data were best fitted with the Langmuir and pseudo-second-order models, (4) Positive value of ∆H° and negative value of ∆G° indicated that AZT adsorption process was spontaneous and endothermic in nature, (5) After 10 reuses of the MIL/Cs@Fe_3_O_4_ NCs, the AZT removal efficiency dropped from 98.36 to 68.12, and (6) The inhibition effect of background electrolytes on AZT adsorption by MIL/Cs@Fe3O4 NCs follows a trend of Mg^2+^ > Ca^2+^ > K^+^ = Na^+^  > NO_3_^−^ > Cl^−^ > SO_4_^2−^ > CO_3_^2−^.

## Supplementary Information

Supplementary Information.

## Data Availability

The data that support the findings of this study are available from the first author, [Ali Azari], on special request. Part of the data and materials are also available within supplementary materials.
