# Author Correction: Magnetic NH_2_-MIL-101(Al)/Chitosan nanocomposite as a novel adsorbent for the removal of azithromycin: modeling and process optimization

**DOI:** 10.1038/s41598-023-38418-w

**Published:** 2023-07-17

**Authors:** Ali Azari, Mohammad Malakoutian, Kamyar Yaghmaeain, Neemat Jaafarzadeh, Nabi Shariatifar, Gholamabbas Mohammadi, Mahmood Reza Masoudi, Reza Sadeghi, Sanaz Hamzeh, Hossein Kamani

**Affiliations:** 1 Sirjan School of Medical Sciences, Sirjan, Iran; 2 Student Research Committee, Sirjan School of Medical Sciences, Sirjan, Iran; 3 Department of Environmental Health Engineering, School of Public Health, Kerman University of Medical Sciences, Kerman, Iran; 4 Department of Environmental Health Engineering, School of Public Health, Tehran University of Medical Sciences, Tehran, Iran; 5 Department of Environmental Health Engineering, School of Public Health, Jondishapour University of Medical Sciences, Ahvaz, Iran; 6 Health Promotion Research Center, Zahedan University of Medical Sciences, Zahedan, Iran

Correction to: *Scientific Reports* 10.1038/s41598-022-21551-3, published online 08 November 2022

The Acknowledgements section in the original version of this Article was incomplete.

“This work was supported by the Zahedan University of Medical Sciences, Zahedan, Iran [Grant No. 10588].”

now reads:

“The authors would like to thank Zahedan University of Medical Sciences for funding this research. (Grant number: 10588, Ethics code: IR.ZAUMS.REC.1401.167).”

The original Article has been corrected.

