# Supplementary material for: Magnetic NH2-MIL-101(Al)/Chitosan nanocomposite as a novel adsorbent for the removal of azithromycin: modeling and process optimization
Source: Sci Rep. 2022 Nov 8;12:18990. doi: 10.1038/s41598-022-21551-3 (PMC9643464; doi:10.1038/s41598-022-21551-3)
Supplement: Supplementary file 1 — Supplementary Information. [file 41598_2022_21551_MOESM1_ESM.pdf]

Supporting Information for

# Magnetic $NH_2$-MIL-101(Al) /Chitosan nanocomposite as a novel adsorbent for the removal of azithromycin: modeling and process optimization

Ali Azari [1, 2], Mohammad Malakoutian [3], Kamyar yaghmaeain [4], Neemat Jaafarzadeh [5], Nabi Shariati Far [4], Gholamabbas Mohammadi [1], Mahmood Reza Masoudi [1], Reza Sadeghi [1], Sanaz Hamzeh[1], Hossein Kamani [6, *]

[1] Sirjan School of Medical Sciences, Sirjan, Iran

[2] Student Research Committee, Sirjan School of Medical Sciences, Sirjan, Iran

[3] Department of Environmental Health Engineering, School of Public Health, Kerman University of Medical Sciences, Kerman, Iran,

[4] Department of Environmental Health Engineering, School of Public Health, Tehran University of Medical Sciences, Tehran, Iran

[5] Department of Environmental Health Engineering, School of Public Health, Jondishapour University of Medical Sciences, Ahvaz, Iran

[6] Health Promotion Research Center, Zahedan University of Medical Sciences, Zahedan, Iran

* **Corresponding author**: Prof. Hossein Kamani,
Phone: +98 9124528677,
Fax: +98 21 88779487,
E-mail address: azari.hjh@gmail.com.

**Supplementary Information, Text 1**

### 2.1.1. Chemicals and reagents

The model antibiotic, Azithromycin (AZT) was supplied by Tehran Pharmaceutical Co, Ltd. (Tehran, Iran). shrimp shells were provided from local markets in Tehran, Iran. $FeCl_2.4H_2O$ (≥99.0%), $FeCl_3.6H_2O$ (≥97.0%), N, N-Dimethylformamide anhydrous (DMF, ≥99.8%), $AlCl_3 \cdot 6H_2O$ (≥99%), Acetone (≥99.5) and 2-aminoterephthalic acid (≥99%) were purchased from Sigma Aldrich (St. Louis, MO, USA) and were of analytical grade. $H_2SO_4$ (>99.0%), HCl (35-37%), and NaOH (93%) were purchased from Merck Co. (Darmstadt, Germany). Deionized water was utilized throughout this research. All other chemicals were of analytical grade and used as received without any further purification.

### 2.1.2. Instrumentation and analytical methods

The stock solution of AZT was produced by dissolving foreordained amounts of AZTs in 1000 mL deionized water, and other required concentrations were prepared through the appropriate dilution of the stock solution. Stock solutions were stored in a dark place at 4 °C (refrigerator). HCl and NaOH 1 N were used to justify pH to desired values. The pH values were measured using a pH meter (Metrohm 744, Switzerland). The separation of synthesized nano-composite from aquatic solution was carried out through a cubic magnet (magnetic field of 3,000 Gauss). The initial and residual concentration of AZT was determined using UV–Visible spectrophotometer at a maximum absorbance wavelength of 482 nm [1].

**Supplementary Information, Text 2**

### 2.2.1. Chitosan obtained from shrimp shell (Cs)

A predetermined amount of shrimp exoskeletons was washed with water, and oven-dried at 80 °C for 4 h. Then, demineralization was carried out by adding 1000 mL of 1 M HCl to 100 g of shrimp shells. The reaction proceeded at 30 °C under agitation at 250 rpm for 2h. Afterwards, the demineralized shells were filtrated and washed with distilled water until neutral pH. Subsequently, the demineralized shrimp shells were ground, and subjected to chemical treatment in 1 M NaOH solution at a solid/liquid ratio of 1:10 (g/mL). Reaction was carried out under agitation with magnetic stirring at 80 °C for 3 h for removal of proteins. The solid was filtrated and washed with distilled water until it achieved neutral pH. Then, it was immersed in ethanol for 10 min for further bleaching, and the resulting chitin was dried in an oven at 80 °C. A discoloration process was made with a solution of 15% ether, 75% acetone, and 10% distilled water at 50 °C for 3 h, with magnetic stirring. Finally, the sample was exhaustively washed with distilled water. The resulting Chitosan was filtrated, washed with distilled water until neutral pH, and dried in an oven at 70°C [2,3].

**Supplementary Information, Text 3**

### 2.3. Characterization

The crystal phases of the samples were determined using an XRD diffractometer (D8 Advance, Bruker, Germany) with Cu Ka radiation (k= 1.540562 A, 40 kV, 40 mA). The relative intensity was measured throughout a scattering range of 5 to 70 degrees (2=5°-70°). The surface morphology and structure of the sorbents were examined using scanning electron microscopy (SEM, Philips XL-30, USA) at 15 kV. The $N_2$ sorption-desorption isotherms were determined at 77 K using a Micromeritics Tristar 3000 analyzer to examine the textural features of samples. The BET and BJH procedures were used to determine the specific surface area and pore size

distribution of materials. Transmission electron microscopy (TEM, Hitachi, H-7500, Japan) operating at 80 kV was used to assess the size distribution and polydispersity of MIL/Cs@$Fe_3O_4$ NCs. Fourier transform infrared spectra (FTIR, Shimadzu-8400S spectrometer, Japan) were utilized to identify the chemical functional groups contained in the materials at wavenumbers ranging from 500 to 4000 $cm^{-1}$. The magnetic characteristics of the adsorbents were measured using a vibrating-sample magnetometer (VSM, Quantum Design MPMS SQUID, USA).

**Table S1.** BET and BJH results of samples

| Samples | Surface area ($m^2/g$) | Average pore Volume ($Cm^3/g$) | Average pore size (nm) |
|---|---|---|---|
| **$Fe_3O_4$ NPs** | 76 | 0.23 | 1.9 |
| **$NH_2$-$MIL_{Al}$ -101** | 306.12 | 0.47 | 2.23 |
| **MIL/Cs NCs** | 448.95 | 0.51 | 2.74 |
| **MIL/Cs@$Fe_3O_4$ NCs** | 451.73 | 0.64 | 2.78 |

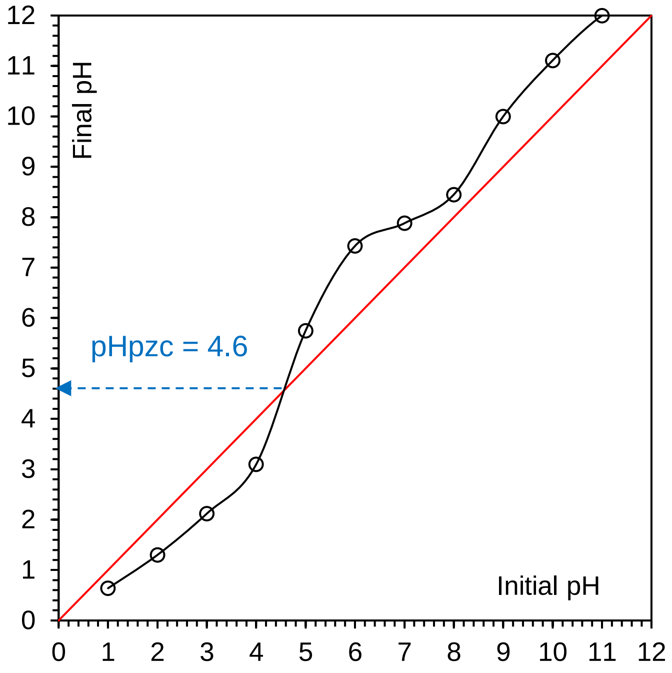


**Fig. S1.** pHpzc of MIL/Cs@$Fe_3O_4$ NCs.

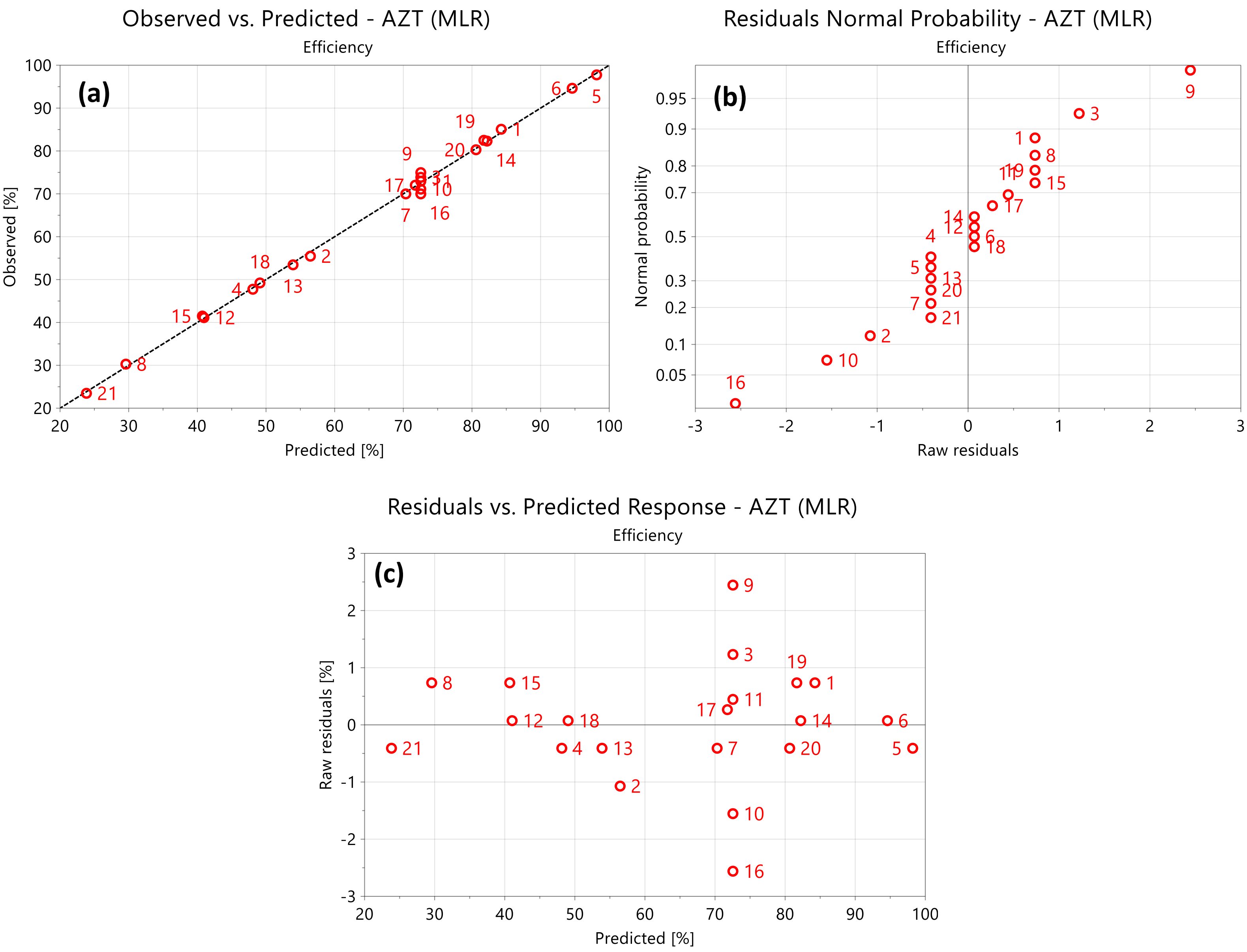


**Fig. S2.** Plots of actual data Vs. predicted data (a) Normal probability of the studentized residuals (b) and distribution of the residuals around the standard line (c).

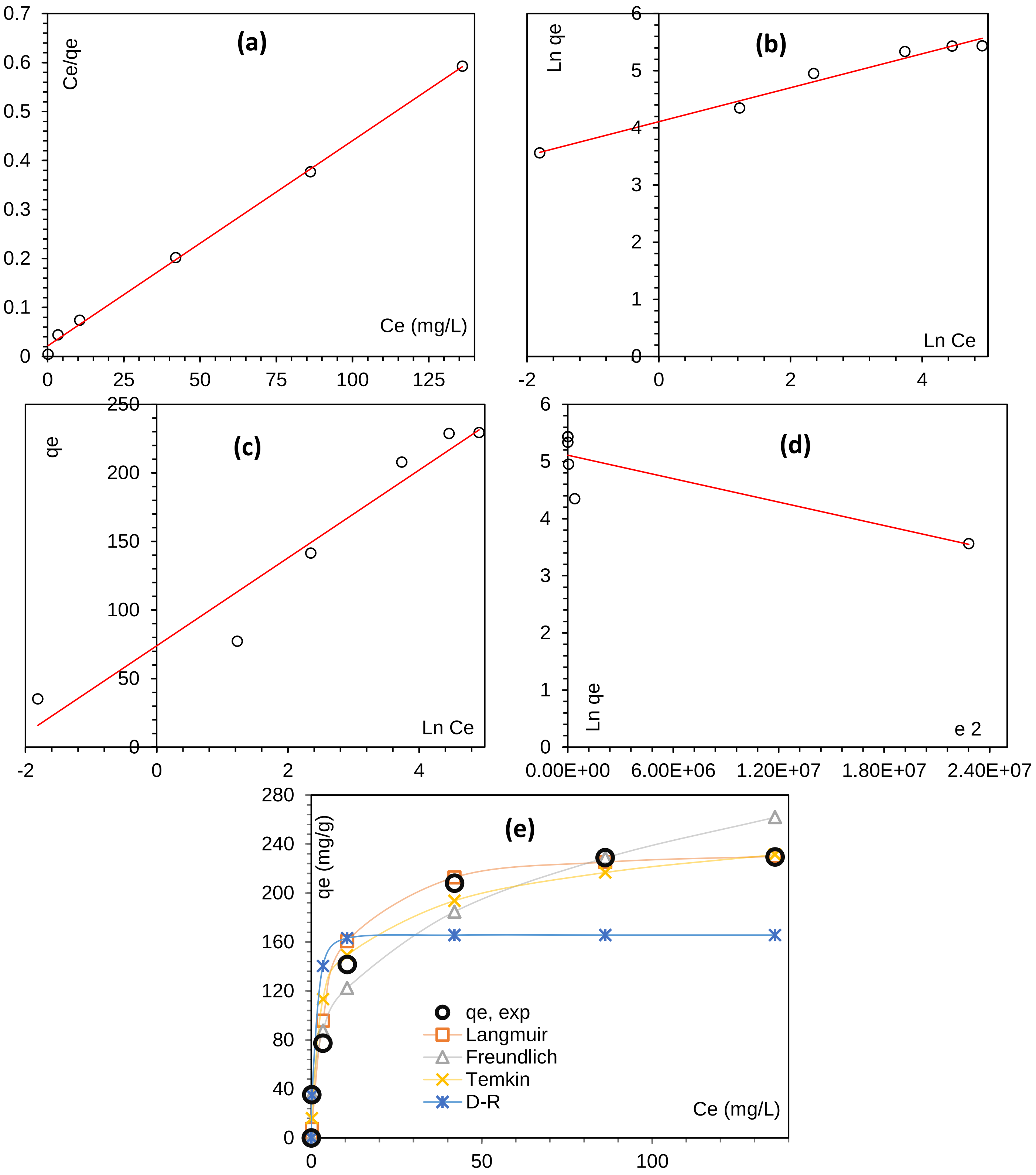


**Fig. S3.** Langmuir (a), Freundlich (b), Temkin (c), and D-R (d) and non-linear isotherm models (e) for AZT adsorption on MIL/Cs@$Fe_3O_4$ NCs under optimized conditions

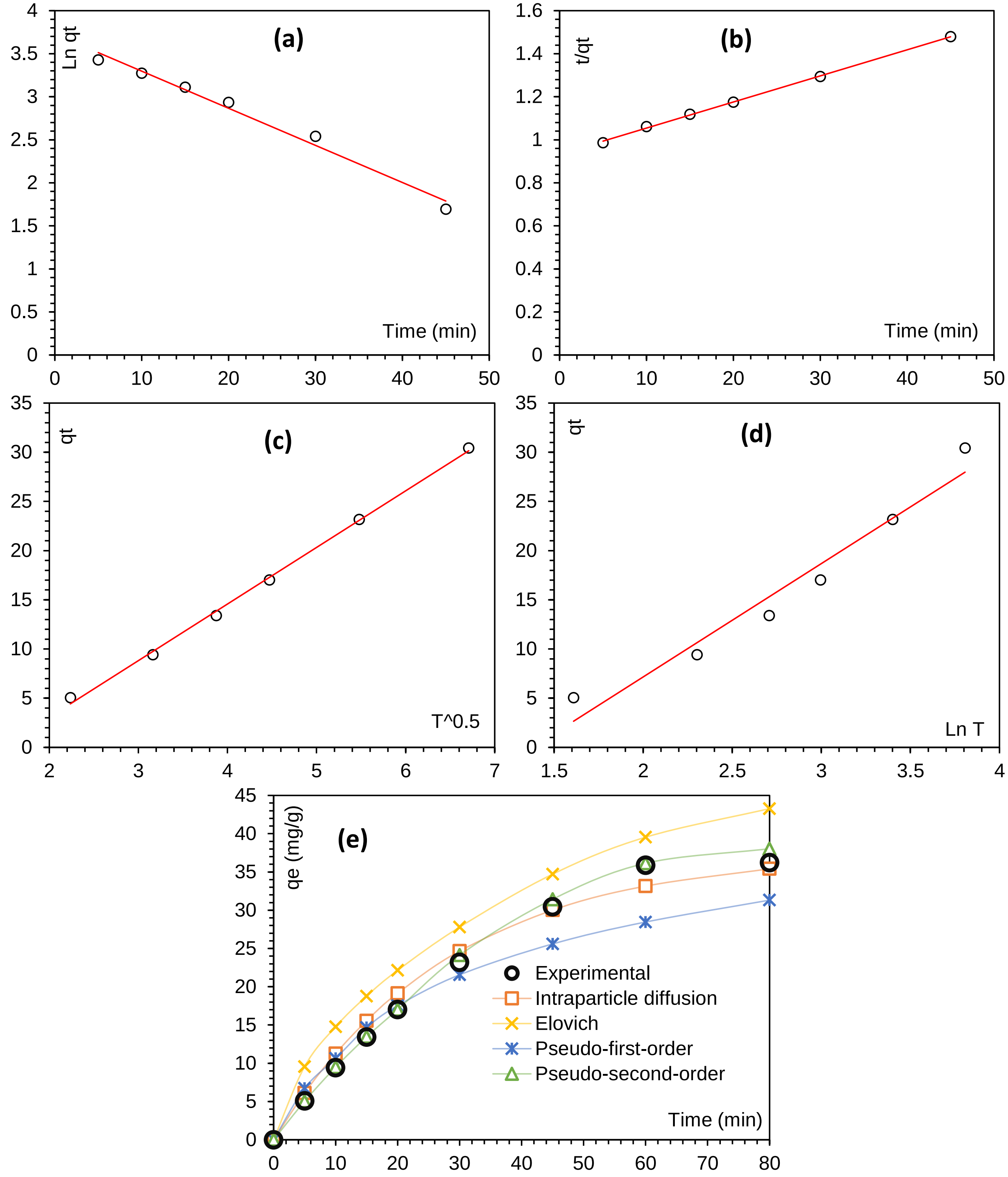


**Fig. S4.** pseudo-first-order (a), pseudo-second-order (b), Elovich (c), intraparticle diffusion (d) and non-linear kinetic models (e) for AZT adsorption on MIL/Cs@$Fe_3O_4$ NCs under optimized conditions

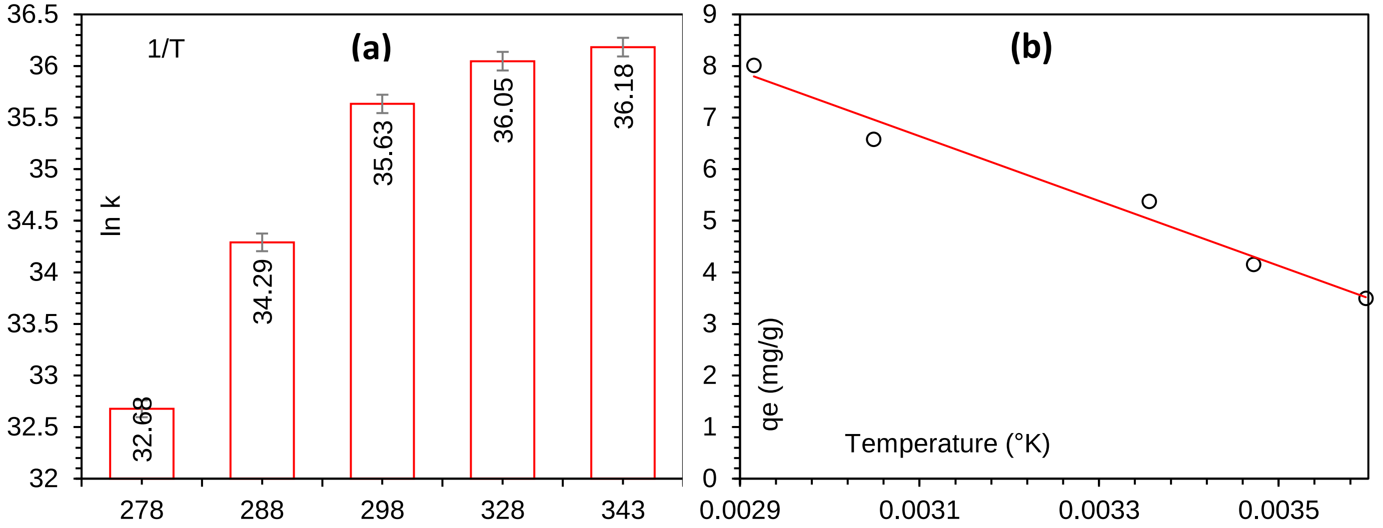


**Fig. S5.** Effect of temperatures on the AZT adsorption(a), thermodynamic Van 't Hoff plots (b)

**Table S2.** The quality of different water matrices.

| Parameters | Tap water | Treated wastewater | Raw wastewater | Surface runoff |
|---|---|---|---|---|
| pH | 7.2 ± 0.1 | 7.1 ± 0.3 | 6.4 ± 0.3 | 6.6 ± 0.2 |
| COD (mg/L) | ND | 171 ± 7 | 264 ± 3 | 205 |
| Turbidity (NTU) | 3 | 5 | 15 | 19 |
| Conductivity (µs/cm) | 985 ± 20 | 877 ± 10 | 1019 ± 12 | 18.3 |

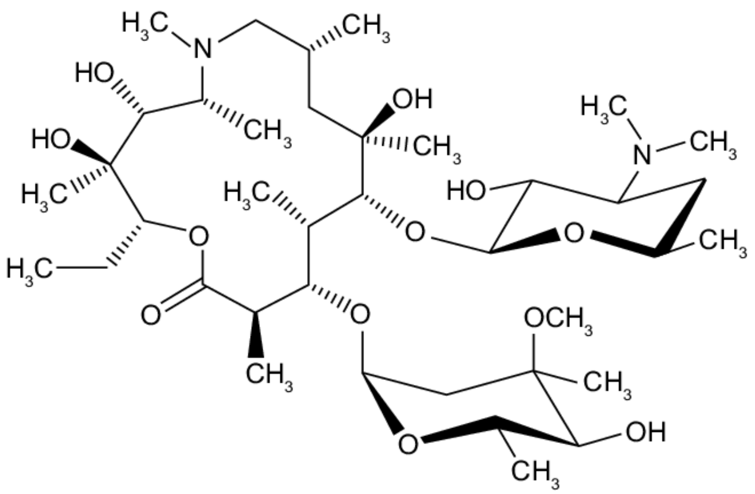

**Fig. S6.** Chemical structure of azithromycin.

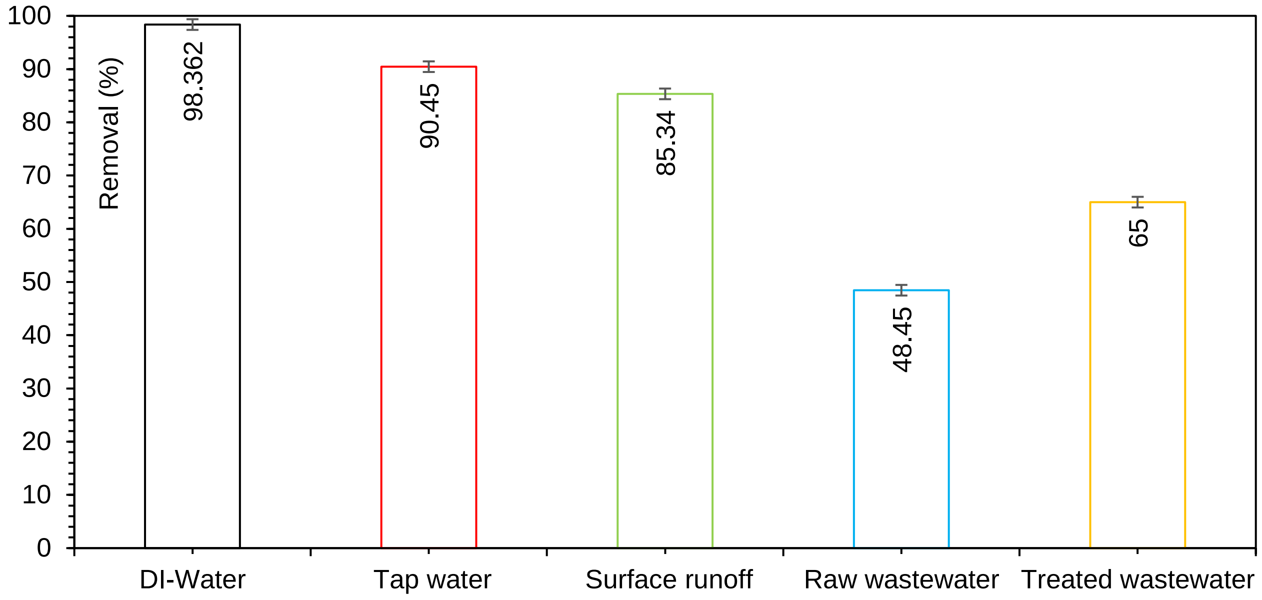


**Fig. S7.** Adsorption efficiency of AZT by MIL/Cs@$Fe_3O_4$ NCs under real conditions.

## Reference:


1 Imanipoor, J., Mohammadi, M. & Dinari, M. Evaluating the performance of L-methionine modified montmorillonite K10 and 3-aminopropyltriethoxysilane functionalized magnesium phyllosilicate organoclays for adsorptive removal of azithromycin from water. *Separation and Purification Technology* **275**, 119256 (2021).

2 Torres-Hernández, Y. G. *et al.* Biological compatibility of a polylactic acid composite reinforced with natural chitosan obtained from shrimp waste. *Materials* **11**, 1465 (2018).

3 De Queiroz Antonino, R. S. C. M. *et al.* Preparation and characterization of chitosan obtained from shells of shrimp (Litopenaeus vannamei Boone). *Marine drugs* **15**, 141 (2017).
